# Multiepitope-Based Peptide Vaccine Against A35R Glycoprotein and E8L Membrane Protein of Monkeypox Virus Using an Immunoinformatics Approach

**DOI:** 10.3390/biology15070524

**Published:** 2026-03-25

**Authors:** Laaiba Attique, Syed Babar Jamal, Tayyaba Gulistan, Adnan Haider, Deeba Amraiz, Sumra Wajid Abbasi, Sajjad Ahmad, Mohammad Abdullah Aljasir

**Affiliations:** 1 Department of Biological Sciences, National University of Medical Sciences, Rawalpindi 46000, Pakistandeeba.amraiz@numspak.edu.pk (D.A.); 2 National Center for Bioinformatics, Quaid-i-Azam University, Islamabad 15320, Pakistan; tayyaba.gulistan93@gmail.com; 3 Department of Health and Biological Sciences, Abasyn University, Peshawar 25000, Pakistan; sajjad.ahmad@abasyn.edu.pk; 4 Department of Medical Laboratories, College of Applied Medical Sciences, Qassim University, Buraydah 51431, Saudi Arabia

**Keywords:** monkeypox virus, multiepitope vaccine, immunoinformatics, A35R glycoprotein, E8L membrane protein, protein–protein docking, molecular dynamic simulation, free-energies estimation

## Abstract

Monkeypox virus, a zoonotic DNA virus belonging to the *Orthopoxvirus* genus, has emerged as a global health issue because of its fast spread to 104 nations over six continents. In the current study, an immunoinformatics pipeline was used to design a multiepitope-based prophylactic vaccine targeting the A35R glycoprotein and E8L membrane proteins of the monkeypox virus. Selected target proteins were surface-exposed, non-homologous to the human proteome, and essential for viral pathogenesis. B-cell and T-cell (MHC-I and MHC-II) epitopes with high antigenicity (>0.5), non-allergenicity, non-toxicity, and highly soluble in water with strong affinity towards innate and adaptive receptors, were prioritized. Shortlisted epitopes were combined to design the final vaccine utilizing an adjuvant (50S ribosomal L7/L12) and appropriate linkers for improved immunogenicity. Population coverage analysis showed wide HLA representation with 83.57% (MHC-I) and 88.8% (MHC-II) global coverage, including 89.6% for West Africa and 87.3% for Central Africa. Docking analysis of the vaccine construct with the TLR-4 receptor revealed stable interactions (−695.6 kcal/mol). Molecular dynamics simulations and binding free energies further confirmed structural stability. Immune simulations predicted strong activation of both humoral and cellular immune responses. These results indicate that the designed multiepitope vaccine construct is a viable option for additional experimental validation against the monkeypox virus.

## Simple Summary

Monkeypox occurrences around the world have sparked concerns about future epidemics. Safe and effective vaccines are essential for protecting people around the world. In this study, an *in silico* strategy was used to develop a novel vaccine against monkeypox, with the goal of addressing the need. We studied important viral proteins found on the virus’ surface that were unrelated to proteins in humans and were needed for the virus to cause infection. A single vaccine design was made by combining carefully chosen epitopes. This can train the body’s immune system to spot the virus and fight it. We also added a natural immunity booster to make the body’s defenses stronger. Current research reveals that the computationally created vaccine is effective not only in monkeypox endemic areas, but also for a larger part of the global population. Mechanistic investigation of the vaccine construct using molecular dynamics simulation also verifies the stability of the vaccine and its potential to strongly stimulate the body’s defense system, resulting in the generation of protective antibodies. Overall, this study offers a promising first step towards designing a vaccine that is both safe and affordable, which may help prevent monkeypox infections and lessen the effects of future outbreaks around the world.

## 1. Introduction

The human monkeypox virus (MPXV) is a linear, double-stranded DNA molecule of approximately 197 kb encoding more than 190 open reading frames (ORFs). The highly conserved genes responsible for the transcription and replication of virus reside on the central region of the genome, while the terminal regions encode proteins involved in host range determination, immune evasion, and virulence [1,2]. MPXV is a member of the genus *Orthopoxvirus*, a family of viruses that includes one of the most complicated and severe animal viruses: variola virus, cowpox virus, and vaccinia virus [2,3]. Monkeypox virus may be traced back to 1958 in Denmark, when it was discovered in monkeys held for research [4]. The first human case of monkeypox virus was documented in 1970 in Basankusu, Democratic Republic of Congo, involving a nine-month-old boy who succumbed to the virus [5]. Since then, the virus has predominantly been documented in endemic regions of West and Central Africa [6]. In 2022, a multi-country outbreak of monkeypox in humans emerged alongside the ongoing coronavirus disease 2019 (COVID-19) pandemic, raising significant concerns over its human-to-human transmissibility, particularly in non-endemic regions [7]. The virus has disseminated to 104 countries across six continents, with the greatest prevalence observed in North America and Europe [1]. Pakistan reported its first confirmed case of monkeypox virus on 21 April 2024, a 25-year-old man returning from Saudi Arabia [8]. This rising worldwide presence highlights the need for improved surveillance and prevention methods [9]. Monkeypox is clinically similar to smallpox, characterized by fever, headache, fatigue, myalgia, and lymphadenopathy, followed by the emergence of synchronous lesions that evolve through macular, papular, vesicular, and pustular stages [10,11,12]. A number of viral membrane and envelope proteins are important targets for vaccine development because they are involved in immune detection and host cell attachment. The currently available vaccine, ACAM2000, originally meant for smallpox, is being repurposed to prevent monkeypox [13]. Despite being vaccinated with ACAM2000, people experience a subsequent attack [14]. The risk of a secondary attack is approximately 9.28% in unvaccinated individuals compared to 1.31% in vaccinated individuals [15]. Moreover, major side effects like myopericarditis, eczema vaccinatum, and progressive vaccinia are linked to ACAM2000 [16]. In light of the adverse effects associated with existing monkeypox vaccines and the rising number of cases worldwide, the development of a new vaccine through reverse vaccinology has become critically important. Reverse vaccinology leverages high-throughput omics data and advanced computational tools to identify potential antigens, which can then be used to design epitope-based or mRNA vaccines. Consequently, using reverse vaccinology techniques, this study attempts to create one of the first computationally designed multi-epitope vaccines that targets both the E8L membrane protein and the A35R glycoprotein proteins simultaneously. Since they are surface associated viral proteins involved in viral attachment, membrane fusion, and host immunological interaction, A35R glycoprotein and E8L membrane protein were chosen as vaccine targets. Further, these proteins are attractive targets for epitope-based vaccine design since they are conserved among orthopoxviruses and are known to contribute to viral virulence and immune evasion mechanisms.

## 2. Material and Methods

**Figure 1:**
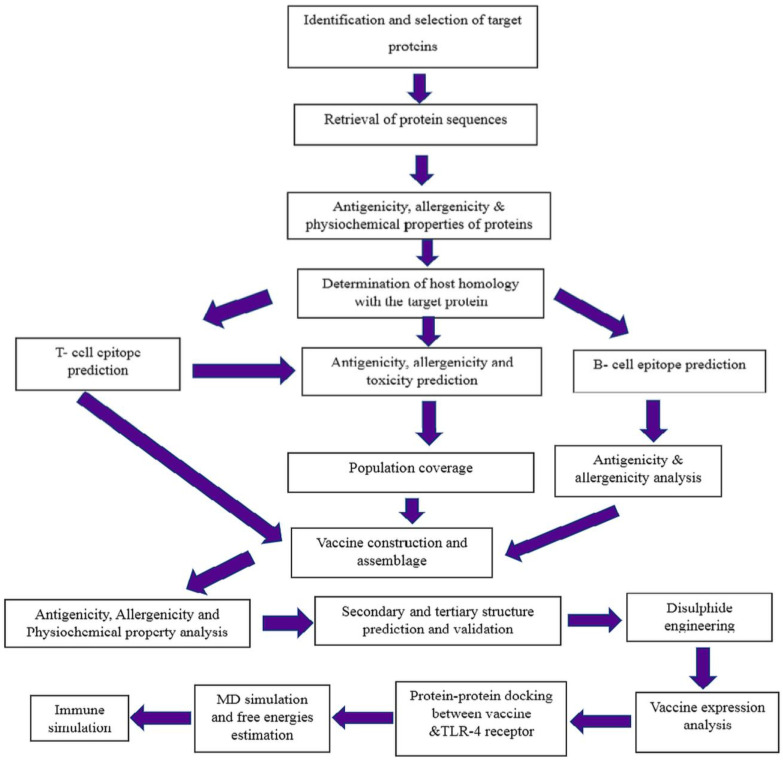
Workflow for reverse vaccinology.

In order to design a multi-epitope vaccine that targets the monkeypox virus’s A35R glycoprotein and E8L membrane protein, a comprehensive *in silico* pipeline was set up (Figure 1).

### 2.1. Vaccine Candidate Selection and Sequence Retrieval

The sequences of the target proteins, E8L membrane protein (Q8V4Y0) and A35R glycoprotein (Q8V4U4), were retrieved in FASTA format from UniProt. The coordinates for the TLR-4 receptor (PDB ID: 4G8A) were retrieved from the Protein Data Bank (https://www.rcsb.org/structure/4G8A, accessed on 23 September 2025).

### 2.2. Antigenicity and Allergenicity Determination of Putative Immunogenic Targets

Vaxijen version 2.0., was used to perform antigenicity analysis of selected proteins (http://www.ddg-pharmfac.net/vaxijen/VaxiJen/VaxiJen.html, accessed on 23 September 2025) [17]. The non-allergic nature of the proteins was confirmed using AllerTOP 2.0 (https://www.ddg-pharmfac.net/AllerTOP/, accessed on 23 September 2025).

### 2.3. Determination of Host Homology with the Target Protein

The target protein’s homology with the host was evaluated using the NCBI’s BLASTp (v2.13.0). For a viral protein to be chosen as an immunogenic target, it should share no more than 30% homology with the host [18].

### 2.4. Physicochemical Properties and Structural Analysis of Putative Immunogenic Targets

ExPAsy Protparam was used to calculate the target proteins’ physicochemical parameters (https://web.expasy.org/protparam/, accessed on 25 September 2025). The PSIPHRED tool (http://bioinf.cs.ucl.ac.uk/psipred/, accessed on 25 September 2025) and the SOPMA server were used to predict the secondary structure of the proteins (https://npsa.lyon.inserm.fr/cgi-bin/npsa_automat.pl?page=/NPSA/npsa_sopma.html, accessed on 25 September 2025). The target protein’s disulfide linkages were predicted using the DiANNA tool (v1.1), which is available at https://bioinformatics.bc.edu/clotelab/DiANNA/, 25 September 2025. On the other hand, the I. TASSER/t-Rosetta tool (https://zhanggroup.org/I-TASSER/, accessed on 25 September 2025) was used to make a prediction about the tertiary structure of the targets.

### 2.5. Prediction and Profiling of B and T-Cell Epitopes

B-cell epitopes are parts of proteins that B-cell receptors or antibodies can directly recognize and start the humoral immune response. Using IEDB Linear Epitope Prediction Tool v2.0, available through the Immune Epitope Database (IEDB), B-cell epitopes were predicted. MHC class I T-cell epitopes were identified using IEDB MHC-I Binding Prediction tool, whereas MHC class II T-cell epitopes were predicted using IEDB MHC-II Binding Predictions Tool (http://tools.iedb.org/mhcii/, accessed on 29 September 2025). Importantly, the projected 9-mer core binding regions generated by the IEDB software coincide with the MHC class II epitopes identified in our work. These core sequences are frequently used for ranking and selecting high-affinity epitopes because they show the binding register within longer peptide segments (typically 12–20 amino acids) involved in MHC class II presentation. The predicted epitopes were further screened for antigenicity and allergenicity using Vaxijen 2.0 server and AllerTOP v2.0, respectively. Toxicity analysis of selected epitopes was conducted via ToxinPred, a canopy to 3595 non-toxic and 1805 toxic peptides [19]. Furthermore, the physicochemical properties for all the shortlisted epitopes, including hydrophobicity, hydrophilicity (GRAVY), charge, isoelectric point (PI), and solubility, were computed using INNOVAGEN PepCalc tool (https://pepcalc.com). IFN-γ positive nature of MHC class II epitopes was evaluated using IFNepitope server (https://bio.tools/ifnepitope, accessed on 29 September 2025).

### 2.6. Population Coverage

Both geographic location and ethnic background can have an effect on the prevalence of different HLA alleles. For the purpose of determining the extent to which the vaccine will provide immunity to different geographical groups, the IEDB population coverage analysis tool (http://tools.iedb.org/population/, accessed on 29 September 2025) was utilized. The MHC I and MHC II epitopes in relation to their respective alleles were submitted to the IEDB population coverage analysis tool.

### 2.7. Multi-Epitope Vaccine Construction and Assemblage

All the MHC I and MHC II binding epitopes along with the shortlisted B-cell epitopes were used for the construction of the multi-epitope vaccine. An adjuvant, 50S ribosomal protein that specializes in increasing the immunogenicity of the vaccine was bound to the vaccine’s N-terminal using the EAAAK linker [20]. EAAAK, GPGPG, AAY and KK are the primary types of linkers that were used in the process of vaccine construction. The EAAAK linker, in addition to its role in creating a helical structure, also enhances the stability of the vaccine construct [21]. Moreover, GPGPG, a glycine-rich linker, accounts for the increased structural integrity and solubility of the vaccine construct [22]. Despite being effective recognition sites for proteosomes to perform their effective cleavage activities, the AAY linkers also play a significant role in preventing junctional immunogenicity [23]. A 6x His tag is inserted at the C terminal of the vaccine construct using RVRR linker.

### 2.8. Solubility and Physicochemical Analysis of Vaccine Construct

The antigenicity of the vaccine construct was estimated using the Vaxijen 2.0 server with a threshold value of 0.4, whereas the allergenicity was evaluated using AllerTOP v2.0. Toxicity analysis was performed using ToxinPred, which employs an ML method based on Support Vector Machines (SVM) and hybrid methods to determine the toxicity of peptides [24]. INNOVAGEN PepCalc was used to find the solubility of the vaccine construct. Physicochemical properties of the vaccine construct were evaluated using the ExPASy-Protparam tool (https://web.expasy.org/protparam/, accessed on 1 October 2025).

### 2.9. Secondary and Tertiary Structure Extrapolation and Validation

The secondary structure of the vaccine construct was predicted using Psipred (http://bioinf.cs.ucl.ac.uk/psipred/, accessed on 1 October 2025). Swiss model was used to design the tertiary structure of the vaccine construct. The generated 3D structure was further optimized using Galaxy Refine to enhance the structural stability (https://galaxy.seoklab.org/cgi-bin/submit.cgi?type=REFINE, accessed on 1 October 2025). Following the refinement, the quality of the generated 3D structure was confirmed using PROCHECK (https://www.ebi.ac.uk/thornton-srv/software/PROCHECK/, accessed on 1 October 2025). PROCHCEK generated a Ramachandran plot which classified the residues in favored, allowed, and disallowed regions. Further, to evaluate the compatibility of the designed structure with the crystallographic structures, ProSA-web was employed (https://prosa.services.came.sbg.ac.at/prosa.php, accessed on 1 October 2025).

### 2.10. Disulfide Engineering

Disulphide bonds are significant covalent interactions which account for the stability of the target protein as well as its interactions and dynamics [25]. In an attempt to increase the stability of the vaccine structure it was submitted to the Disulfide by Design (DbD) tool version 2.12 (http://cptweb.cpt.wayne.edu/DbD2/, accessed on 22 March 2026). To select potential residue pairings, a set of parameters (87 toþ97 chi3 value and 2.2 energy value) was used, which was then mutated with a cysteine residue.

### 2.11. Vaccine Expression Analysis

Protein to nucleotide translation of the vaccine was performed using EMBOSS v 6.5.7. The codons were optimized in accordance with the host organism using Jcat available at https://www.jcat.de. An unadaptive or unoptimized codon is indicative of trifling expression rate. Amplification and *in silico* cloning of the nucleotide sequence were performed using the pET-28a(+) expression vector to check the expression potential of the vaccine construct. To put the vaccine construct’s optimized gene sequence into the vector, NdeI and XhoI restriction sites were used. Then, SnapGene software was used to show how *in silico* cloning works (https://www.snapgene.com).

### 2.12. Protein–Protein Docking Analysis

Protein–protein interaction studies were conducted using Cluspro v2.0 (https://cluspro.bu.edu/login.php, accessed on 5 October 2025). Cluspro makes use of a Fourier correlation algorithm [26]. This algorithm works by taking in coordinates of two independent, distinct, crystalline structures of proteins and creating a complex. Cluspro rotates the macromolecules using a predefined list consisting of 13,000 rotations; as a result, 2.7 × 10^10^ structures are generated [27]. A total of 20,000 structures possessing the best surface complementarity scores are retained. They are then passed through various stages such as filtration, clustering, and ranking to generate the best binding poses [28].

### 2.13. Molecular Dynamics Simulation

Molecular dynamics simulation analysis of the vaccine construct with the TLR-4 receptor was performed using AMBER 22 [29]. MD simulation was performed using a TIP3P water box with the ff14SB force field. The process began by neutralizing the systems with Na^+^ and Cl^−^ atoms followed by energy minimization of all the systems. The systems were brought to equilibrium for 100 ps under the NVT and NPT ensembles, with temperature and pressure set to no more than 300 K and 1 atm. The graphs were then plotted to quantify various parameters such as the RMSD, RMSF, Rg and Beta factor.

### 2.14. Free Energies Estimation

The free energies estimation of the system was performed using the MM-PBSA method which relies upon the solution of two equations for calculating the free energies of the systems, namely Generalized Born equation and Poisson–Boltzmann equation. MM-PBSA method works by comparing the free energy of the same molecule in two different solvated conformations where most of the energy contribution comes from solvent–solvent interactions [30].

### 2.15. Immune Simulations

The immunological simulation was carried out using the C-ImmSim server (https://150.146.2.1/C-IMMSIM/index.php, accessed on 15 October 2025). The server performed *in silico* immunological simulations to assess the immunogenicity of the MPXV vaccine and the immune response it produces.

## 3. Results

### 3.1. Antigenicity and Allergenicity Determination of Putative Immunogenic Targets

The selected proteins were evaluated for their antigenicity and allergenicity. The antigenicity scores for A35R glycoprotein and E8L membrane protein were 0.5 and 0. 5316, respectively. Both proteins were found to be non-allergenic and suitable for vaccine development. While some epitopes were near the 0.4–0.5 threshold, they were retained because they were highly conserved across orthopoxviruses and mapped to regions of functional importance.

### 3.2. Determination of Host Homology with the Target Protein

The vaccine was developed to be used in humans; it is extremely important to ensure that the selected viral proteins do not possess homology to the host proteins. Thus, to evaluate host homology, BLASTp analysis against the human proteome was done, utilizing the NCBI database. The results obtained confirmed the target proteins’ suitability for vaccine development, as no significant homology to human proteins was observed (Figure S1).

### 3.3. Physicochemical Properties and Structural Analysis of Putative Immunogenic Targets

The ExPAsy Protparam computer program was used to predict the physicochemical properties of the target proteins. The A35R glycoprotein was found to be composed of 33.70% alpha helices, 18.78% extended strands and 47.51% coils, whereas the E8L membrane protein was composed of 28.62% alpha helices, 20.72% extended strands and 50.66% coils. The parameters were set to default values (window width: 17; similarity threshold: 8; and number of states: 3). Sequences were submitted in the FASTA format to determine the number of disulphide bonds. The number of disulphide bonds predicted by DiANNA in A35R protein was six at various locations: Cys36-cys62, Cys100-Cys109, and Cys126-Cys180. In the E8L protein, we found less than two cysteines; therefore, the disulphide bond could not be formed. The tertiary structure of the target proteins was determined using trRosetta. The most favorable models (model 1) were selected for both the target proteins. Model 1 for A35R had an accuracy of 99.7%, identity of 86.2, E-value of 1.2 × 10^−19^ and a Z score of 25.319, whereas Model 1 for the E8L membrane protein had a very high confidence level with a coverage of 76.3%, identity with the model structure of 34.7%, E-value of 2 × 10^−55^ and Z-score of 20.463. A summary of the physicochemical and structural properties of the target proteins is provided in Table S1.

### 3.4. Prediction and Profiling of B and T-Cell Epitopes

Five B-cell epitopes from A35R glycoprotein were identified and evaluated for their antigenicity and allergenicity. All five epitopes had antigenicity scores above 0.4. The highest antigenicity score was 0.7299 for epitope STLPNKSDVL, and the lowest score was 0.4611 for epitope ENDEEQTSVFSATVYGDKIQGKNKR. Then, all the selected epitopes were assessed for allergenicity using AllerTOP v2.0. Four out of five were non-allergenic and one epitope, ENDEEQTSVFSATVYGDKIQGKNKR, was identified as a possible allergen. For E8L membrane protein we identified twelve B-cell epitopes; four epitopes with less than eight residues were eliminated. Antigenicity analysis of the remaining eight epitopes revealed the highest antigenicity score of 0.7119 for epitope DSIRSANMSAPF. The lowest antigenicity score was 0.2187 for epitope YLDNLLPSTL. This was followed by allergenicity analysis. The shortlisted epitopes were both antigenic and non-allergenic in nature. Table 1 and Table 2 represent the selected B-cell epitopes for A35R and E8L glycoprotein with their position in the protein sequence, length and antigenicity score.

After the identification of B-cells, MHC class 1 T-cell epitopes were identified. For two antigenic proteins the MHC class I epitopes were found using the IEDB MHC 1 binding prediction tool. Forty-three epitopes from A35R glycoprotein and eighty-seven epitopes from E8L membrane proteins were identified to have an IC-50 value smaller than 50. The epitopes from both proteins were evaluated for their antigenicity and allergenicity. Thirty epitopes had an antigenicity score above 0.4, whereas 13 epitopes could not reach the desired threshold for antigenicity. Fifteen epitopes from A35R and 25 epitopes from E8L were competent to fulfill the antigenicity threshold and were found to be non-allergenic at the same time. We further evaluated the epitopes for their rank > 1, toxicity, hydrophobicity, hydrophilicity (GRAVY), charge, isoelectric point (PI), and solubility. Consequently, seven epitopes from A35R and five epitopes from E8L were found to be antigenic, nontoxic, non-allergenic with good water solubility. Supplementary Tables S2 and S3 represent the predicted MHC class I T-cell epitopes, along with their binding affinities and immunological characteristics for A35R (Table S2) and E8L proteins (Table S3). The A35R glycoprotein and E8L membrane protein were further scrutinized for MHC class II T-cell epitopes. Seventeen epitopes from A35R and 25 epitopes from E8L were antigenic and non-allergenic. Furthermore, the epitopes were evaluated for further characteristics such as rank > 10, IFN-γ toxicity, hydrophobicity, hydrophilicity (GRAVY), charge, isoelectric point (PI), and solubility. Consequently, five epitopes from A35R and seven epitopes from E8L were found to possess these characteristics. Table 3 and Table 4 represent the predicted MHC class II T-cell epitopes for A35R (Table 3) and E8L proteins (Table 4).

### 3.5. Population Coverage

**Figure 2:**
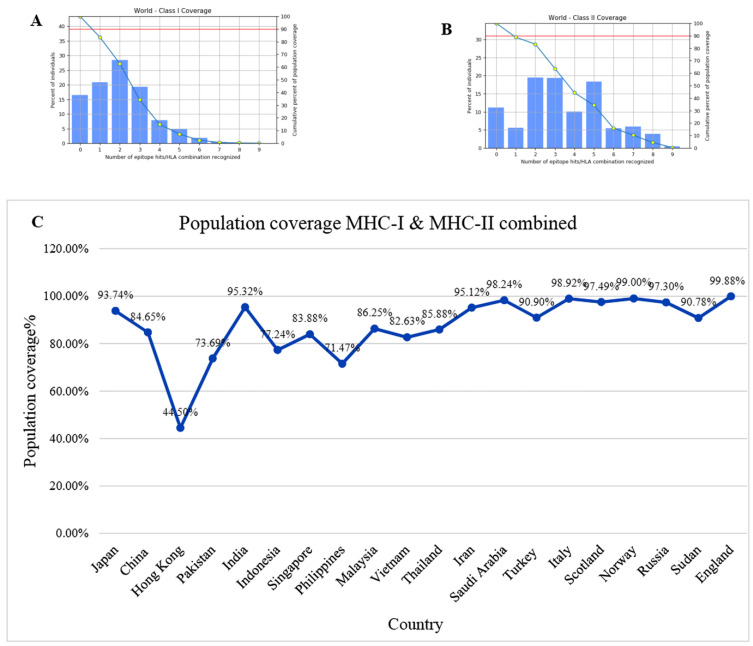
(**A**) World population coverage of MHC class I epitopes. (**B**) World population coverage of MHC class II epitopes. (**C**) Population coverage peaks for twenty countries across the globe.

A total of twelve MHC I binding epitopes extracted from both the target protein along with their corresponding alleles were submitted to IEDB MHC I population coverage tool. The following alleles were found to have MHC class I T-epitopes: HLA-A*01:01, HLA-A*02:01, HLA-A*02:06, HLA-A*30:02, HLA-A*31:01, HLA-A*32:01, HLA-A*33:01, HLA-A*68:01, HLA-B*15:01, HLA-B*35:01, HLA-B*44:02, HLA-B*44:03 and HLA-B*58:01. The world population coverage graph for MHC class I is shown in Figure 2A. A population coverage of 83.57% with an average hit of 2.06 and PC90 of 0.61 was attained. Population coverage was performed for MHC class II T-cell epitopes with their corresponding alleles. MHC class II T-cell epitopes with their corresponding alleles HLA-DQA1*01:02/DQB1*06:02, HLA-DPA1*01:03/DPB1*02:01, HLA-DPA1*01:03/DPB1*04:01, HLA-DPA1*02:01/DPB1*01:01, HLA1*03:01/DPB1*04:02, HLA-DRB1*01:01, HLA-DRB1*03:01, HLA-DRB1*04:01, HLA-DRB1*04:04, HLA-DRB1*04:05, HLA-DRB1*07:01, HLA-DRB1*08:02, HLA-DRB1*09:01, HLA-DRB1*11:01, HLA-DRB1*12:01, HLA-DRB1*13:01, HLA-DRB1*13:02, HLA-DRB1*15:01, HLA-DRB1*16:02, HLA-DRB3*01:01, HLA-DRB3*02:02, HLA-DRB3*03:01, HLA-DRB4*01:01, HLA-DRB4*01:03, and HLA-DRB5*01:01 were submitted to IEDB MHC-II population coverage tool. The tool predicted a population coverage of 88.8% with an average hit of 3.46 and PC90 of 0.89, as shown in Figure 2B. Figure 2C shows the population coverage of MHC class I and class II combined for 20 countries. The graph represents the highest population coverage of 99.88% for England, followed by Norway with 99% and Italy with 98.92%.

### 3.6. Multiepitope Vaccine Construction and Assemblage

**Figure 3:**
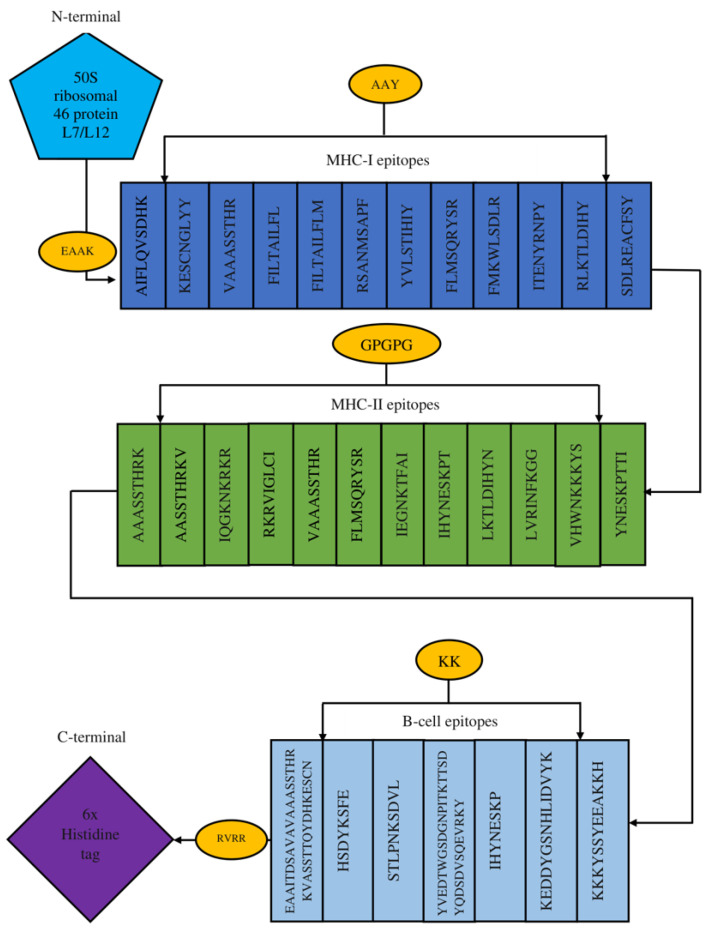
Diagrammatic representation of vaccine construct.

The multi-epitope-based vaccine construct was formed using MHC-I, MHC-II and B-cell epitopes extracted from the A35R glycoprotein and E8L membrane protein. The epitopes were joined using primary linkers, and an adjuvant, 50S ribosomal protein L7/L12 was added [31]. Figure 3 shows the diagrammatic representation of the vaccine construct representing the B-cell epitopes along with MHC class I and class II epitopes. The EAAAK linker was used to join E8L ribosomal protein with MHC-I epitopes, the AAY linker was used to join MHC I epitopes together, the GPGPG linker joined MHC II epitopes, the KK linker was used to join B-cells epitopes and the RVRR linker was used to join B-cell epitopes with Histidine tag, as represented by Figure 3.

### 3.7. Solubility and Physicochemical Analysis

The antigenicity of the vaccine construct was found to be 0.5614, at the threshold of 0.4. The allergenicity estimation of the vaccine construct revealed that it was a probable non-allergen. The physicochemical properties represented in Table 5 demonstrate, amongst various parameters, a stability index of 36.43, which represents our vaccine construct being stable along with having good water solubility.

### 3.8. Extrapolation and Confirmation of Secondary and Tertiary Structures

**Figure 4:**
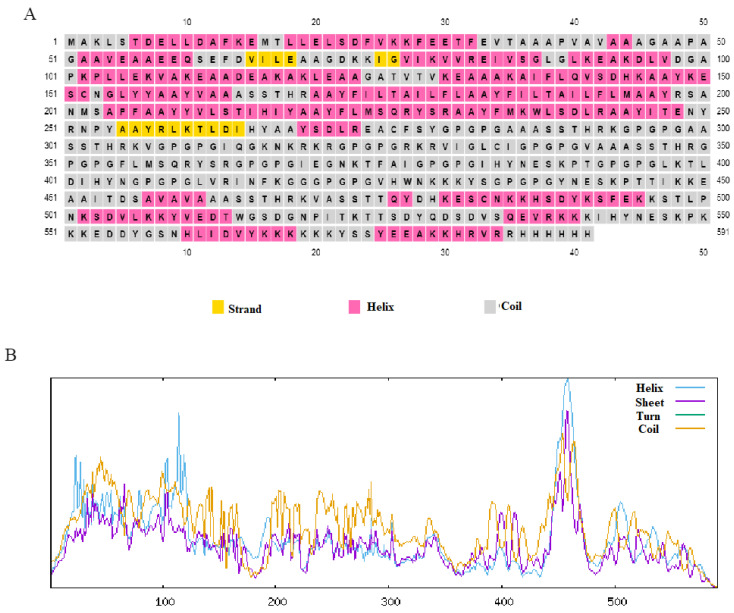
(**A**) Secondary structure composition by Psiphred. (**B**) Peaks representing the various secondary structure components of vaccine construct obtained using SOPMA server.

The final vaccine construct was found to have 591 amino acids. It was submitted to Psipred in the FASTA format for secondary structure prediction. The structural composition of the vaccine construct was further analyzed using two diverse tools, PSIPRED and SOPMA. Figure 4A represents the secondary structure composition obtained using Psiphred, whereas 4B represents the peaks depicting secondary structure components obtained using the SOPMA server.

**Figure 5:**
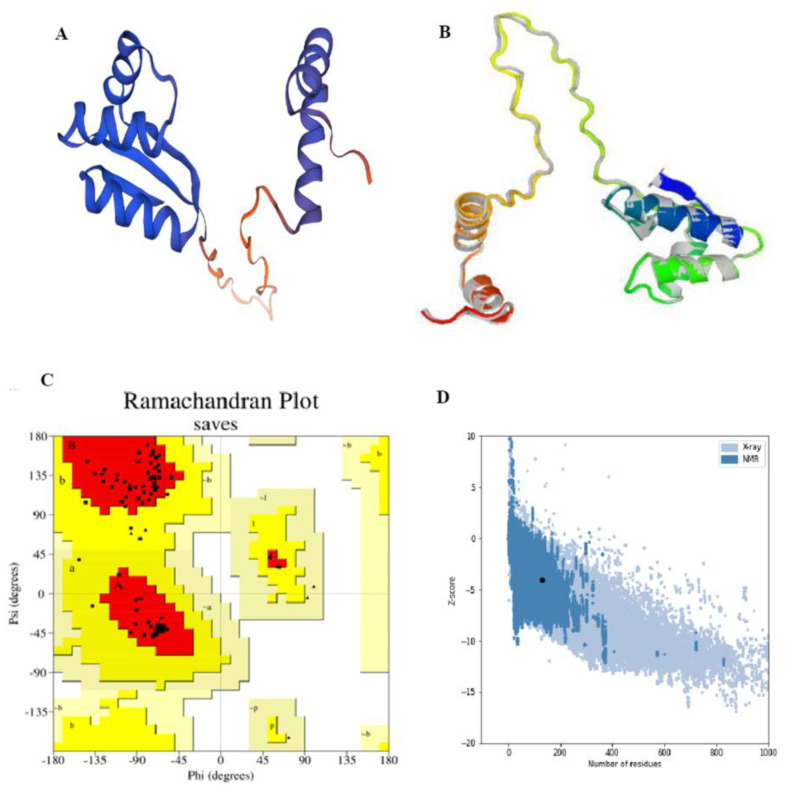
(**A**) Tertiary structure of the vaccine predicted by Swissmodel. (**B**) Refined energy model of vaccine obtained by GalaxyRefine. (**C**) Ramachandran plot represents the positions of various interacting residues from most-favored regions to disallowed regions. (**D**) Plot obtained from ProSA-web server indicating overall model quality of the vaccine.

The Swissmodel server was used to predict the three-dimensional structure of the vaccine construct. The server predicted five models, among which model 2 had 100% sequence identity with the template structure and a GMQE score of 0.17 (Figure 5A). The corresponding PDB file of the selected model was submitted to the Galaxy Refine server to refine the tertiary structure of the protein. Galaxy Refine generated five refined models based on parameters like GDT-HA, clash score, RMSD, poor rotamers, MolProbity, and Rama-favored regions. Table 6 represents the values of these characteristics for the initial model as well as the five refined models. Among the generated models, model 2 was selected as the best, having GDT-HA of 0.9712, clash score of 2.6, RMSD of 0.360, poor rotamers of 1.1, MolProbity of 1.331, and Rama-favored region of 96.1. A low value of RMSD is indicative of high quality of structure, compactness of residues, and less deviation. Figure 5B represents the tertiary structure of Model 2 obtained after refinement.

The refined model generated from GalaxyRefine was further validated using PROCHECK to generate the Ramachandran plot (Figure 5C). Ramachandran plot reveals the presence of 95.7% of the residues in most favored regions, 4.3% in additional allowed regions, 0.0% in generously allowed and 0.0% in disallowed region. The efficiency of the ProSA-web server was also utilized to evaluate the quality of the model. The plot represented by Figure 5D revealed a Z-score of −4.1, which strongly represents low energy and strong structural stability.

### 3.9. Disulfide Engineering

Disulfide engineering was performed to increase the structural stability of the vaccine construct by introducing a cysteine bond. The intra- and inter-structural chains of the proteins are carefully scrutinized for residues which are capable of being mutated to add cysteine bonds. Consequently, ten pairs of residues were identified as potential candidates. The residue pairs with their location, energy and Chi3 angles are summarized in Table 7.

### 3.10. Vaccine Expression Analysis

**Figure 6:**
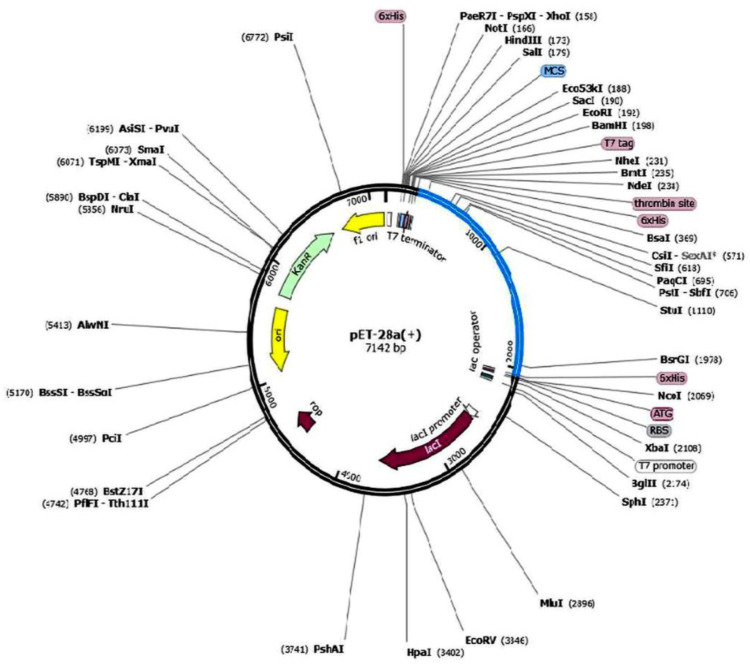
The mutant form of pET-28a (+) vector generated for *in silico* cloning of vaccine.

The vaccine construct consisted of 591 amino acids that were reverse-transcribed into 1784 nucleotides for codon optimization. After reverse transcription of the protein sequence, the sequence was submitted to the Jcat server for codon adaptation and expression optimization. Sequence obtained from EMBOSS had a CAI of 0.90957 and a GC content of 66.949. After the optimization of nucleotide sequence by the JAVA Codon Adaptation tool (Jcat), the CAI value was increased to 0.9560 and the GC content was increased to 68.3023. The initial sequence was purified and all the invalid nucleotides were removed. For amplification and *in silico* cloning, the optimized nucleotide sequence of the vaccine consisting of 1773 bp was inserted into the pET-28a (+) vector using the SnapGene software version 5.7.1. The mutant form of the vector is shown in Figure 6.

### 3.11. Protein–Protein Docking Analysis

**Figure 7:**
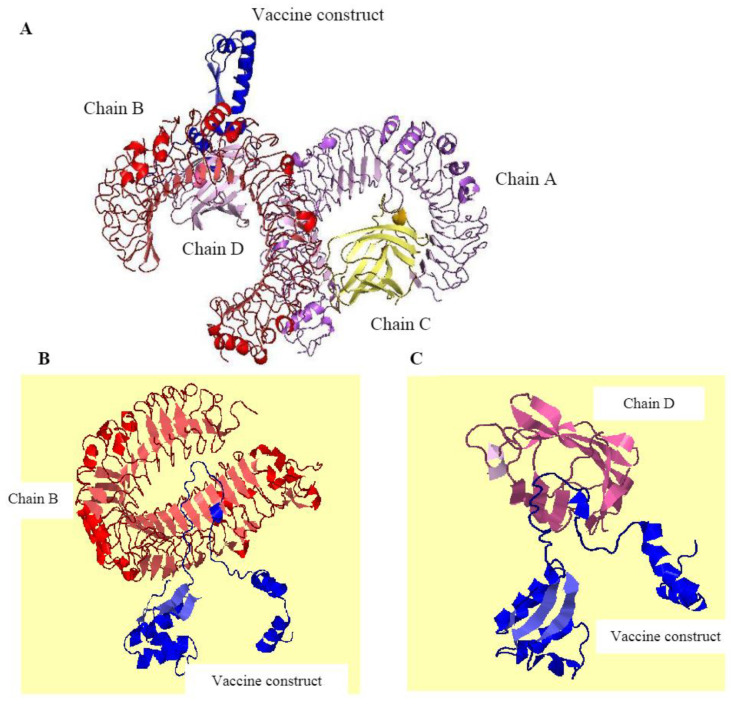
(**A**) The binding interaction of the TLR-4 receptor (indicated by chains A, B, C and D) with the vaccine construct. (**B**) Binding interaction of chain B with the vaccine construct during molecular docking. (**C**) Binding interaction of chain D with the vaccine construct during molecular docking.

Protein–protein docking is crucial in several pathophysiological processes to predict the possible interactions of antigenic viral protein with the immune receptors [32]. In the current study, protein–protein docking interaction was performed between the vaccine and TLR-4 innate receptor. TLR-4 was chosen as the docking receptor due to its significant role in innate immune recognition and the activation of downstream immune signaling pathways that promote adaptive immune responses. The pdb file of the TLR-4 receptor was downloaded from the PDB using ID 4G8A and was docked with the refined structure of the vaccine using Cluspro. Figure 7A represents the interaction of TLR-4 receptor with the vaccine construct. The visual examination of the docked complex demonstrated that the designed construct interacted predominantly with chains B and D, which facilitated the principal binding interface of the receptor–vaccine complex. Chains A and C, on the other hand, did not show any significant interactions with the vaccine construct and stayed outside the main interaction region (Figure 7B,C). For the 167 Ǻ interface area on chain B and 223 Ǻ on the vaccine construct, a total of 20 non-bonded contacts were found between Arg87, Glu89, Phe63 and Glu42 residues of chain B and Ala50 and Pro49 residues on the vaccine construct. Moreover, 120 non-bonded interactions were found between chain D of the receptor and the vaccine construct. The interacting residues on chain D included Pro43, Leu154, Ser31, Asn26, Trp23, Val24, Tyr34, Ile44, Tyr22, Ser45, Lys20, Ile46, Asn47, Phe64, Asn114, Asn49 and Pro50, whereas, on the vaccine construct, the interacting residues were Ala48, Gly46, Glu33, Val34, Thr35, Ala37, Ala38, Pro39, Ala43, Ala44, Val42, Ala45, Ala41, Ala52, Ala53, Val54 and Glu59. Cluspro generated 26 models depicting the TLR-4-construct binding interaction with the binding energies and the number of interacting partners. All the models of molecular docking were keenly scrutinized and Model 0 for the vaccine-TLR-4 interaction was selected based on the minimum value of weighted score (−695.6), as well as the maximum number of interacting partners, which was 53.

### 3.12. Molecular Dynamic Simulations

**Figure 8:**
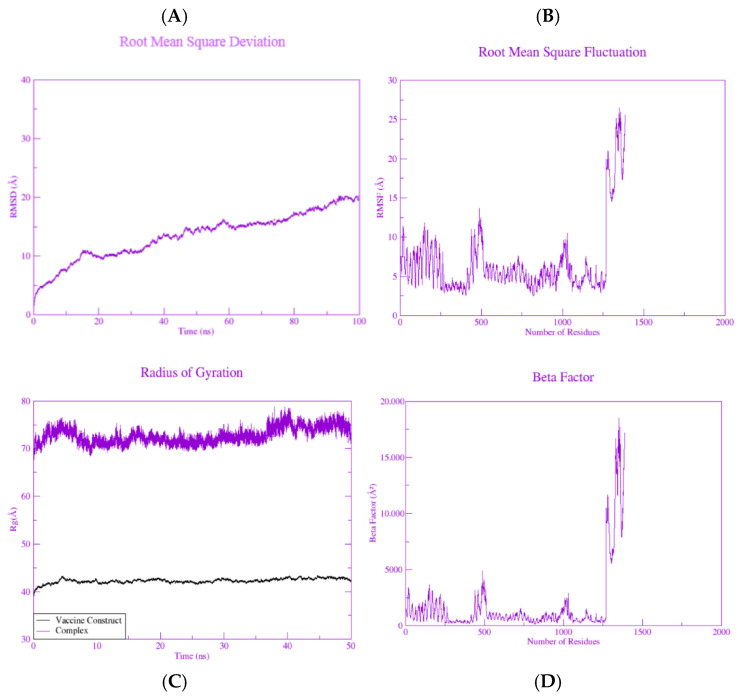
(**A**) MD simulation trajectory for RMSD represents a steadily increasing value. (**B**) MD simulation trajectory for RMSF showing fluctuations in the interacting atoms of vaccine-TLR-4 complex. (**C**) MD simulation trajectory representative of the radius of gyration. (**D**) Representation of the Beta factor vs. number of residues representative of atomic positional fluctuations.

Molecular dynamic simulations of the vaccine-TLR-4 docked complex were performed using AMBER 22 to evaluate not only the vaccine’s stability but also its structural adjustments when it binds to the innate receptor (TLR-4). A 100 ns production run was performed to check the structural and functional stability. Time-dependent statistical parameters such as the root mean square deviation (RMSD), root mean square fluctuation (RMSF), surface accessibility and radius of gyration (Rg) were calculated throughout simulation run. To determine the conformational stability of the vaccine-TLR-4 complex, a RMSD vs. time graph was plotted (Figure 8). The RMSD gradually increased during the simulation and eventually stabilized, indicating structural equilibration of the system. The mean RMSD value was 13.59 Å, with a minimum RMSD of 0 Å at frame 0 and a maximum RMSD of 15.15 Å at frame 8992. The relatively high RMSD value was primarily due to flexible loop regions, which are expected in multi-epitope constructs [33]. Importantly, the core regions involved in receptor binding remained stable, suggesting the overall structural integrity was preserved. The compactness of the protein structure was evaluated using the radius of gyration (Rg). The Rg profile of the system remained consistent with the RMSD, suggesting stable structural compactness throughout the simulation. The mean Rg of the construct was observed to be 42.2719 Å, whereas the maximum Rg of 43.342 Å with standard deviation of 0.492 Å was observed. The TLR-4-vaccine complex had an average Rg value of 72.70 Å, with a maximum value of 78.78 Å. The mechanics of the system and residual flexibility were studied by plotting a graph of RMSF against the number of residues. The mean RMSF for the system was found to be 6.8 Å, highlighting moderate fluctuations. Fluctuations are often found in vaccine constructs because they are composed of numerous loops with no fixed structure. The loops play a significant role for presenting the vaccine construct to the immune system. RMSF was followed by the evaluation of the β-factor, which completely complemented the RMSF. For the β-factor, a mean value of 694 was noted, whereas the maximum value of the β-factor remained 1387. Figure 8 represents the RMSD, RMSF and radius of gyration and the B-factor of the TLR-4-vaccine complex.

### 3.13. Free Energies Estimation

MM-PBSA, a highly efficient program for estimating the free energies of the system’s molecules, utilizes the trajectories generated as a result of MD simulations. Different energy components along with their binding free energies are tabulated in Table 8, Table 9, Table 10 and Table 11. The net energies for GB and PB were estimated to be −82.7535 kcal/mol and −25.6967 kcal/mol, which were highly significant for the system. In GB, a delta energy of −29,520.2191 kcal/mol for the complex, 27,844.3289 kcal/mol for the receptor and −1593.1367 kcal/mol for the vaccine construct was estimated (Table 8). The maximum contribution to the delta energy in GB was made by the vaccine construct (Table 9).

For PB, a delta energy of −24,525.7324 kcal/mol was found for the complex, −23,084.9637 kcal/mol for the TLR-4 receptor and −1415.0720 kcal/mol for the vaccine construct (Table 10). The highest contribution to PB was made by the vaccine construct (−1415.0720 kcal/mol), followed by −23,084.9637 kcal/mol energy of the receptor and 24,525.7324 kcal/mol energy of the complex (Table 11). As far as the electrostatic energy of the system is concerned, the value remained the same for both GB and PB. An electrostatic contribution of −1006.8938 kcal/mol was estimated for the system. Considering the individual electrostatic contribution, the vaccine construct (−8074.6901 kcal/mol) was found to contribute the most to net PB in comparison to the complex (−96,018.9505) and TLR-4 receptor (−88,951.1543). The next energy component was van der Waal energy, which favored the system’s total free energy. The van der Waal energy (GB and PB) was found to be −117.1181 kcal/mol for the system on the whole, −9401.5174 kcal/mol for the complex, −8725.4004 kcal/mol for TLR-4 the receptor and −558.9988 kcal/mol for the vaccine construct. The maximum contribution to van der Waal energy for both GB and PB was made by the vaccine construct. Moreover, the net solvation free energy was −1013.4648 kcal/mol for GB and −956.4079 kcal/mol for PB, which is highly favorable to the total energy of the systems. This is greatly attributed to the system’s polar energy, which is −997.4740 kcal/mol for GB and −1025.3507 kcal/mol for PB. The non-polar solvation is −15.9907 kcal/mol for GB and −90.5210 kcal/mol for PB.

### 3.14. Immune Simulations

**Figure 9:**
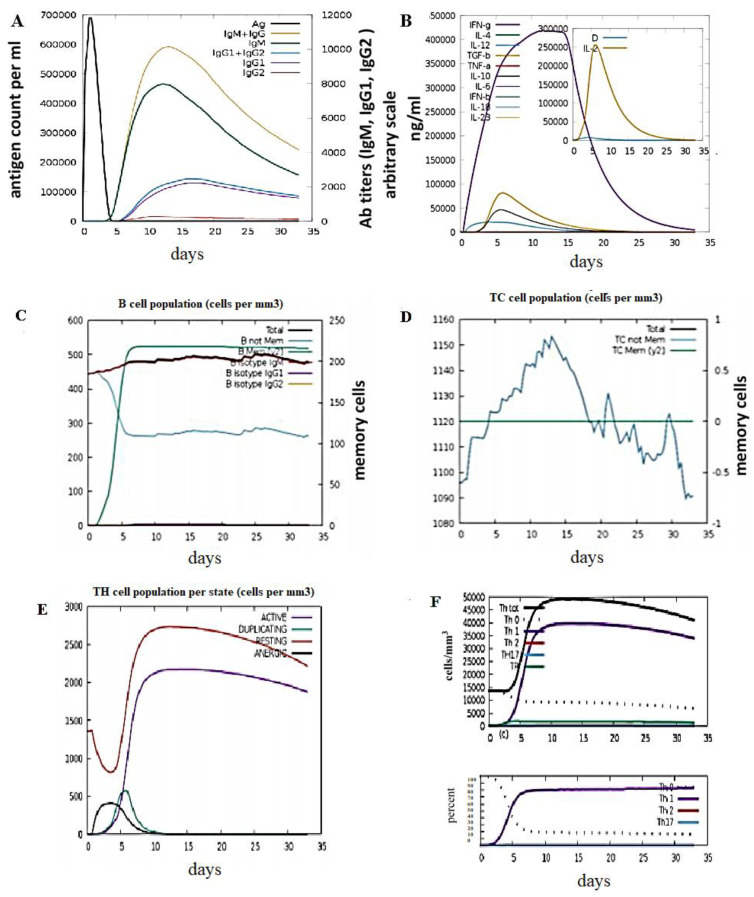
(**A**) Titers of immunoglobulins produced in response to multi-epitope-based vaccine. The Immunoglobulin have been divided based upon their isotypes. (**B**) The levels of cytokines induced after presenting the immune system with the vaccine. The D score represents the Simpson index that is indicative of the dominant T-cell clones produced in response to the vaccine. (**C**) The population of B-cells produced in response to the vaccine represented by B isotype IgM, IgG1 and IgG2. (**D**) The population of cytotoxic T-cells; the maximum response is observed on day 13. (**E**) Population of active, duplicating, resting and energic Th cells produced in response to the vaccine. (**F**) Populations of various distinct types of Helper T-cells.

The C-ImmSim server that employs an agent-based method was utilized to predict the immune responses [34]. The parameters were set to default values, the speed was kept at 12,345, simulation volume at 10, and simulation steps at 100. A total of four alleles—A MHC class I A0101 allele, B MHC class I B0702 allele, and DR MHC class II DRB1_0101 allele—were selected for host HLA selection. The injection’s time step was selected to be 1, whereas the number of antigens to be injected was selected to be 1000. C-ImmSim mimics the immune responses produced in the body by using the vaccine as an antigen. The immune response of the vaccine construct after it was presented to the immune system is shown in Figure 9A. IgM + IgG titers started producing on the 5th day of inoculation and the maximum response was observed at day 15. Figure 9B represents the graph for the cytokine level. The increasing population of B-cells, Helper and cytotoxin T-cells along with Th0, Th1, Th2 and Th17 cells, represents the highly immunogenic nature of vaccine.

## 4. Discussion

The existence of the human race has always been threatened by the presence of disease-causing micro-organisms that are causative agents of a wide spectrum of human and animal diseases. The rapid growth of MPXV around the world in recent years has made it evident that effective means to combat it must be implemented immediately. Vaccination is now the sole appropriate strategy for controlling viral outbreaks and disease spread. Although vaccines originally developed for smallpox, such as ACAM2000 and Modified Vaccinia Ankara (MVA), provide some level of cross-protection against monkeypox, these vaccines are associated with safety concerns and adverse effects [3,13,15]. As a result, it has become a priority to create vaccine strategies that are safer and more focused. Reverse vaccinology and immunoinformatics-based vaccine design have become powerful tools to speed up the discovery of vaccines by finding possible antigenic determinants with the help of computers [23,35]. The aforementioned techniques have a high success rate when applied to a variety of viral infections, including SARS-CoV-2, the influenza virus, and the Ebola virus [22,23,24]. These techniques resulted in the quick identification of immunogenic epitopes that were capable of eliciting both humoral and cellular immune responses with minimal associated risks, unlike traditional vaccine development methods. Multi-epitope vaccines, particularly, have received a lot of attention for their ability to improve immune recognition and widen immune protection by integrating many antigenic determinants into a single construct [22,23].

In the current study, we used the same strategy to develop a multi-epitope vaccine targeting monkeypox virus’s two critical proteins, A35R glycoprotein and E8L membrane protein. The identified proteins are surface-associated proteins that are essential for virus entry and interaction with the host, making them suitable targets for vaccine development because they are accessible through immune surveillance [1,36]. The strong immunogenic potential and lack of allergic characteristics were confirmed by antigenicity and allergenicity tests. The number of B-cell and T-cell epitopes capable of eliciting both humoral and cellular immune responses was determined. The chosen epitopes offer extensive HLA representation with more than 80% expected global coverage according to a population coverage study [37]. The investigation of docked complexes by molecular docking, dynamics simulations, and binding free energy analysis suggests that the complex may trigger immunological signaling in addition to providing additional structural and functional stability [38]. Furthermore, a robust stimulation of the B- and T-cell responses was predicted utilizing immunological models. Overall, the current study’s findings suggest that the suggested multi-epitope vaccine could be a promising candidate for future experimental validation against the monkeypox virus.

Several studies have already identified multi-epitope vaccines against the monkeypox virus; however, the majority of these were based on a single viral protein, whereas the current study reported a construct made up of immunogenic epitopes form A35R and E8L, two vital proteins of monkeypox, which is believed to have improved efficacy as vaccine and also provide broader immune coverage [39,40]. Even though the computational results were quite promising, this study has certain limitations. Computational predictions are primarily algorithm-driven and rely on databases, which may or may not completely cater to the complexity of biological systems and natural processes. Therefore, experimental validation using in vitro immunological tests and in vivo animal studies will be required to verify the immunogenicity, safety, and protective effectiveness of the suggested vaccine candidate.

## 5. Conclusions

This study presents a computational, multi-epitope-based *in silico* vaccine against the monkeypox virus using A35R glycoprotein and E8L membrane protein. The selected proteins are highly antigenic and immunogenic surface proteins that are extremely crucial for the survival of the virus. This vaccine has been developed using linear, highly immunogenic, antigenic, non-allergenic, non-toxin and non-host homologous epitopes, indicating their potential to induce both humoral and cellular immune responses. The secondary and tertiary structure of the vaccine was determined using fully automated, homology-modeling-based server. The molecular docking and dynamics simulation analysis further supported the stability of the final vaccine construct and TLR-4 complex. These results indicate that the proposed multi-epitope vaccine may be a promising candidate for eliciting protective immune responses against MPXV infection. Still, in vitro and in vivo studies need to be done to confirm the safety, immunogenicity, and protective efficacy of the proposed vaccine construct.

## Figures and Tables

**Table 1:** Selected B-cell epitopes for A35R protein.

Selected B-Cell Epitopes for A35R Protein:					
No.	Start	End	Peptide	Length	Antigenicity
1	67	101	EAAITDSAVAVAAASSTHRKVASSTTQYDHKESCN	35	0.6612
2	113	120	HSDYKSFE	8	0.5871
3	131	140	STLPNKSDVL	10	0.7299
4	147	178	YVEDTWGSDGNPITKTTSDYQDSDVSQEVRKY	32	0.4725

**Table 2:** Selected B-cell epitopes for E8L membrane protein.

Selected B-Cell Peptides for E8L Protein					
No.	Start	End	Peptide	Length	Antigenicity
1	26	33	IHYNESKP	8	0.4582
2	72	86	KEDDYGSNHLIDVYK	15	0.4109
3	98	110	KKKYSSYEEAKKH	13	0.4493

**Table 3:** Selected MHC class II T-cell epitopes for A35R protein with IC-50 value, rank, antigenicity, allergenicity, toxicity, hydrophobicity, GRAVY, PI, solubility and IFN-γ positivity.

A35R MHC-II T-Cell Epitopes											
Allele	Peptide	IC 50	Rank	Antigenicity	Allergenicity	Toxicity	Hydrophobicity	GRAVY	PI	Solubility	IFN-γ
HLA-DRB3*03:01	AAASSTHRK	3.7	0.15	1.0499	Non-Allergen	non-toxic	0.44	−0.94	pH 11.42	Good water solubility	Positive
HLA-DRB3*03:01	AASSTHRKV	5.6	0.6	0.7509		non-toxic	4.4	−0.68	pH 11.42	Good water solubility	Positive
HLA-DRB1*13:01 HLA-DRB1*10:01 HLA-DRB4*01:03 HLA-DRB3*03:01 HLA-DRB1*04:04	IQGKNKRKR	10.5	0.93	1.3442		non-toxic	3.58	−2.62	pH 12.18	Good water solubility	Positive
HLA-DRB1*03:01 HLA-DRB1*07:01 HLA-DRB1*15:01 HLA-DRB1*08:02 HLA-DRB1*11:01 HLA-DRB3*01:01 HLA-DRB1*04:04	RKRVIGLCI	43.5	1.8	1.4027		non-toxic	27.25	0.69	pH 11.21	Good water solubility	Positive
HLA-DRB1*04:01 HLA-DRB1*04:04 HLA-DRB1*07:01 HLA-DRB1*16:02 HLA-DRB1*03:01	VAAASSTHR	59.5	2.2	0.6752		non-toxic	3.54	−0.04	pH 10.81	Good water solubility	Positive

**Table 4:** Selected MHC class II epitopes for E8L protein with IC-50 value, rank, antigenicity, allergenicity, toxicity, hydrophobicity, GRAVY, PI, solubility and IFN-γ positivity.

E8L MHC-II T-Cell Epitopes											
Allele	Peptide	IC-50	Rank	Antigenicity	Allergenicity	Toxinpred	Hydrophobicity	GRAVY	PI	Innovagen (Solubility)	IFN-γ
HLA-DRB1*12:01 HLA-DRB3*01:01 HLA-DQA1*01:02/DQB1*06:02 HLA-DRB1*04:05 HLA-DRB1*04:01 HLA-DPA1*01:03/DPB1*02:01 HLA-DRB3*02:02	FLMSQRYSR	15.2	0.6	0.9843	Non-allergen	non-toxic	22.26	−0.77	pH 10.96	Good water solubility	Positive
HLA-DRB1*04:01 HLA-DQA1*01:02/DQB1*06:02 HLA-DRB1*08:02	IEGNKTFAI	53.4	1.9	0.4966		non-toxic	21.97	0.18	pH 6.86	Good water solubility	Positive
HLA-DRB1*07:01 HLA-DRB3*02:02	IHYNESKPT	20.7	2.3	0.5389		non-toxic	11.37	−1.56	pH 7.75	Good water solubility	Positive
HLA-DRB1*01:01 HLA-DRB1*11:01 HLA-DRB1*03:01	LKTLDIHYN	11.3	3.4	1.4807		non-toxic	22.71	−0.44	pH 7.74	Good water solubility	Positive
HLA-DPA1*03:01/DPB1*04:02 HLA-DRB1*07:01 HLA-DRB4*01:01	LVRINFKGG	29.8	3.5	2.006		non-toxic	23.46	0.29	pH 11.41	Good water solubility	Positive
HLA-DRB1*15:01 HLA-DRB1*13:02 HLA-DRB1*11:01 HLA-DRB1*09:01	VHWNKKKYS	38.7	4.9	1.138		non-toxic	17.4	−1.91	pH 10.57	Good water solubility	Positive
HLA-DRB1*07:01 HLA-DRB1*13:02 HLA-DRB1*01:01 HLA-DRB5*01:01	YNESKPTTI	27.5	9.2	0.477		non-toxic	15.74	−1.28	pH 6.57	Good water solubility	Positive

**Table 5:** Physicochemical properties of vaccine construct using protparam.

Physicochemical Properties	
Number of residues	591
Molecular weight	63,853.77g/mol
Extinction coefficient	60,590 M^−1^cm^−1^
Iso-electric point	pH 9.92
Net charge at pH 7	29.9
Estimated solubility	Good water solubility
Instability Index	36.43 (stable)
Aliphatic Index	69.66
Grand Average of Hydrophobicity (GRAVY)	−0.484
Total number of negatively charged residues (Asp + Glu)	58
Total number of positively charged residues (Arg + Lys)	86
Estimated half-life in mammalian reticulocytes	30 h
Estimated half-life in yeast (in vivo)	>20 h
Estimated half-life in E coli (in vivo)	>10 h

**Table 6:** Five refined energy models obtained from GalaxyRefine along with the initial values.

Model	GDT-HA	RMSD	MolProbity	Clash Score	Poor Rotamers	Rama Favored
Initial	1.0000	0.000	1.709	1.0	2.1	85.2
MODEL 1	0.9558	0.398	1.253	2.6	1.1	96.9
MODEL 2	0.9712	0.360	1.331	2.6	1.1	96.1
MODEL 3	0.9654	0.383	1.311	2.6	0.0	96.1
MODEL 4	0.9538	0.395	1.310	3.1	1.1	96.9
MODEL 5	0.9635	0.375	1.504	4.7	0.0	96.1

**Table 7:** Predicted residues for disulphide engineering.

Res1 Chain	Res1 Seq #	Res1 AA	Res2 Chain	Res2 Seq #	Res2 AA	Chi3	Energy	Sum B-Factors
A	16	MET	A	20	GLU	101.69	3.4	0
A	62	GLU	A	107	LYS	−114.74	5.44	0
A	63	PHE	A	113	ALA	88.26	2.7	0
A	65	VAL	A	105	LEU	103.37	4.24	0
A	68	GLU	A	126	THR	125.47	6.14	0
A	69	ALA	A	126	THR	90.94	3.74	0
A	71	GLY	A	124	GLY	80.06	3.31	0
A	73	LYS	A	124	GLY	−62.53	3.34	0
A	76	GLY	A	123	ALA	106.83	2.55	0
A	108	VAL	A	112	ALA	101.7	4.31	0
A	120	LEU	A	125	ALA	100.78	7.13	0

**Table 8:** Generalized Bond energy components calculated using ‘LCPO’ surface area. All values are calculated as kcal/mol.

**GENERALIZED BOND**	**COMPLEX**			
	**Energy Component**	**Mean**	**Average**	**Std. Dev**
	BOND	8.0096	4142.4287	56.6363
	ANGLE	9.7490	11,160.3993	68.9361
	DIHED	6.0381	17,936.5754	42.6962
	VDWAAL	6.1736	−9401.5174	43.6541
	EEL	19.5308	−96,018.9505	138.1038
	VDW	4.0447	4769.7679	28.6001
	EGB	12.3064	−22,146.4698	87.0193
	ESURF	0.3451	610.0073	2.4403
	G gas	20.5066	−7983.7565	145.0037
	G solv	12.1839	−21,536.4625	86.1529
	Total	15.4287	−29,520.2191	109.0976
	**RECEPTOR**			
	**Energy Component**	**Mean**	**Average**	**Std. Dev**
	BOND	7.2801	3850.1091	51.4779
	ANGLE	9.7917	10,331.3114	69.2377
	DIHED	5.5793	16,692.5262	39.4517
	VDWAAL	6.1906	−8725.4004	43.7745
	EEL	17.7435	−88,951.1543	125.4658
	VDW	3.9607	4408.0732	28.0063
	EGB	11.3927	−18,553.5241	80.5584
	ESURF	0.3152	562.0282	2.2289
	G gas	19.3843	−9852.8330	137.0676
	G solv	11.2688	−17,991.4959	79.6824
	Total	14.4156	−27,844.3289	101.9334

**Table 9:** Generalized Bond energy components calculated using ‘LCPO’ surface area. All values are calculated as kcal/mol.

**GENERALIZED BOND**	**VACCINE CONSTRUCT**			
	**Energy Component**	**Mean**	**Average**	**Std. Dev**
	BOND	2.2483	292.1620	15.8981
	ANGLE	3.0752	828.2164	21.7453
	DIHED	1.7740	1236.7199	12.5440
	VDWAAL	1.4777	−558.9988	10.4490
	EEL	4.4042	−8074.6901	31.1424
	VDW	1.0378	359.9745	7.3382
	EGB	3.4364	−2595.4717	24.2990
	ESURF	0.0802	63.9698	0.5672
	G gas	4.7038	938.3652	33.2611
	G solv	3.4061	−2531.5019	24.0851
	Total	4.1694	−1593.1367	29.4823
	**DIFFERENCES COMPLEX-RECEPTOR-VACCINE CONSTRUCT**			
	**Energy Component**	**Mean**	**Average**	**Std. Dev**
	BOND	0.0343	0.1576	0.2429
	ANGLE	0.0915	0.8714	0.6470
	DIHED	0.1039	7.3293	0.7345
	VDWAAL	0.8265	−117.1181	5.8444
	EEL	3.2732	1006.8938	23.1451
	VDW	0.0846	1.7202	0.5979
	EGB	2.8974	−997.4740	20.4878
	ESURF	0.0456	−15.9907	0.3222
	Delta G gas	2.9970	930.7112	21.1922
	Delta G solv	2.9026	−1013.4648	20.5246
	Delta Total	0.5527	−82.7535	3.9085

**Table 10:** Poisson–Boltzmann energy components calculated using internal PBSA solver. All values (kcal/mol) have been represented for complex and receptor.

**POISSON–BOLTZMANN**	**COMPLEX**			
	**Energy Component**	**Mean**	**Average**	**Std. Dev**
	BOND	8.0096	4142.4287	56.6363
	ANGLE	9.7490	11,160.3993	68.9361
	DIHED	6.0381	17,936.5754	42.6961
	VDWAAL	6.1736	−9401.5174	43.6541
	EEL	19.5308	−96,018.9505	138.1038
	VDW	4.0447	4769.7679	28.6001
	EPB	10.8852	−20,907.9464	76.9702
	ENPOLAR	1.8623	11,416.6274	13.1682
	G gas	20.5066	−7983.7565	145.0037
	G solv	10.4118	−16,541.9758	73.6226
	Total	16.1279	−24,525.7324	114.0417
	**RECEPTOR**			
	**Energy Component**	**Mean**	**Average**	**Std. Dev**
	BOND	7.2801	3850.1091	51.4779
	ANGLE	9.7917	10,331.3114	69.2377
	DIHED	5.5793	16,692.5262	39.4517
	VDWAAL	6.1906	−8725.4004	43.7745
	EEL	17.7435	−88,951.1543	125.4658
	VDW	3.9607	4408.0732	28.0063
	EPB	10.1567	−17,305.0271	71.8188
	ENPOLAR	1.7735	10,536.5515	12.5406
	G gas	19.3843	−9852.8330	137.0676
	G solv	9.8732	−13,232.1307	69.8139
	Total	15.2370	−23,084.9637	107.7418

**Table 11:** Poisson–Boltzmann energy components calculated using internal PBSA solver. All values (kcal/mol) have been represented for vaccine construct and complex–receptor–vaccine construct.

**POISSON–BOLTZMANN**	**VACCINE CONSTRUCT**			
	**Energy Component**	**Mean**	**Average**	**Std. Dev**
	BOND	2.2483	292.1620	15.8981
	ANGLE	3.0752	828.2164	21.7453
	DIHED	1.7740	1236.7199	12.5440
	VDWAAL	1.4777	−558.9988	10.4490
	EEL	4.4042	−8074.6901	31.1424
	VDW	1.0378	359.9745	7.3382
	EPB	3.5343	−2577.5686	24.9913
	ENPOLAR	0.4753	970.5968	3.3611
	G gas	4.7038	938.3652	33.2611
	G solv	3.5946	−2353.4372	25.4173
	Total	4.7546	−1415.0720	33.6201
	**DIFFERENCES IN COMPLEX–RECEPTOR–VACCINE CONSTRUCT**			
	**Energy Component**	**Mean**	**Average**	**Std. Dev**
	BOND	0.0343	0.1576	0.2429
	ANGLE	0.0915	0.8714	0.6470
	DIHED	0.1039	7.3293	0.7345
	VDWAAL	0.8265	−117.1181	5.8444
	EEL	3.2732	1006.8938	23.1451
	VDW	0.0846	1.7202	0.5979
	EPB	3.0531	−1025.3507	21.5887
	ENPOLAR	0.2704	−90.5210	1.9123
	DELTA G gas	2.9970	930.7112	21.1922
	DELTA G solv	3.1005	−956.4079	21.9240
	DELTA Total	0.8078	−25.6967	5.7122

## Data Availability

All datasets analyzed in this study are publicly available under the accession numbers stated in the publication; processed data can be obtained from the corresponding author on reasonable request.

## Supplementary Materials

The following supporting information can be downloaded at https://www.mdpi.com/article/10.3390/biology15070524/s1. Table S1. Physicochemical and Structural properties of target proteins. Table S2. Selected MHC Class-I T-cell epitopes for A35R protein with IC-50 value, rank, antigenicity, allergenicity, toxicity, hydrophobicity, GRAVY, PI and solubility. Table S3. Selected MHC Class-I epitopes for E8L protein with IC-50 value, rank, antigenicity, allergenicity, toxicity, hydrophobicity, GRAVY, PI and solubility. Figure S1. Multiple sequence alignment of A35R and E8L proteins generated using Clustal Omega (v 1.2.4).

## References

[1] Harapan H., Ophinni Y., Megawati D., Frediansyah A. (2022). Monkeypox: A comprehensive review. Viruses.

[2] Petersen E., Kantele A., Koopmans M., Asogun D., Yinka-Ogunleye A., Ihekweazu C., Zumla A. (2019). Human monkeypox: Epidemiologic and clinical characteristics. Infect. Dis. Clin. N. Am..

[3] Johri N., Kumar D., Nagar P., Maurya A., Vengat M., Jain P. (2022). Clinical manifestations of human monkeypox infection and implications for outbreak strategy. Health Sci. Rev..

[4] Halder S.K., Sultana A., Himel M.K., Shil A. (2025). Monkeypox: Origin, transmission, clinical manifestations, prevention, and therapeutic options. Interdiscip. Perspect. Infect. Dis..

[5] Beer E.M., Rao B.V. (2019). A systematic review of the epidemiology of human monkeypox outbreaks and implications for outbreak strategy. PLoS Negl. Trop. Dis..

[6] Tajudeen Y.A., Oladipo H.J., Muili A.O., Ikebuaso J.G. (2023). Monkeypox: A review of a zoonotic disease of global public health concern. Health Promot. Perspect..

[7] Ebede S.O., Orabueze I.N., Maduakor U.C., Nwafia I.N., Ohanu M.E. (2025). Recurrent mpox: Divergent virulent clades and the urgent need for strategic measures including novel vaccine development to sustain global health security. BMC Infect. Dis..

[8] Fareed A., Hussain A., Faraz F., Siblini R. (2024). First case of mpox in Pakistan: What can we learn from it?. Health Sci. Rep..

[9] Umair M., Salman M. (2024). Looming threat of mpox in Pakistan: Time to take urgent measures. J. Infect..

[10] Ogoina D., Iroezindu M., James H.I., Oladokun R., Yinka-Ogunleye A., Wakama P., Otike-Odibi B., Usman L.M., Obazee E., Aruna O. (2020). Clinical course and outcome of human monkeypox in Nigeria. Clin. Infect. Dis..

[11] Adler H., Gould S., Hine P., Snell L.B., Wong W., Houlihan C.F., Osborne J.C., Rampling T., Beadsworth M.B.J., Duncan C.J.A. (2022). Clinical features and management of human monkeypox: A retrospective observational study in the UK. Lancet Infect. Dis..

[12] Ciccarese G., Drago F., Parodi A. (2023). Two cases of monkeypox virus infection without detectable cutaneous or mucosal lesions. Travel Med. Infect. Dis..

[13] Keckler M.S., Salzer J.S., Patel N., Townsend M.B., Nakazawa Y.J., Doty J.B., Gallardo-Romero N.F., Satheshkumar P.S., Carroll D.S., Karem K.L. (2020). IMVAMUNE and ACAM2000 provide different protection against disease when administered post-exposure in an intranasal monkeypox challenge prairie dog model. Vaccines.

[14] Abdelaal A., Serhan H.A., Mahmoud M.A., Rodriguez-Morales A.J., Sah R. (2022). Preventing the next pandemic: Is live vaccine efficacious against monkeypox, or is there a need for killed virus and mRNA vaccines?. Vaccines.

[15] Sah R.M., Paul D.M., Mohanty A.M., Shah A.M., Mohanasundaram A.S., Padhi B.K. (2023). Monkeypox vaccines and their side effects: The other side of the coin. Int. J. Surg..

[16] Katamesh B.E., Madany M., Labieb F., Abdelaal A. (2023). Monkeypox pandemic containment: Does the ACAM2000 vaccine play a role in current outbreaks?. Expert Rev. Vaccines.

[17] Doytchinova I.A., Flower D.R. (2007). VaxiJen: A server for prediction of protective antigens, tumour antigens and sub-unit vaccines. BMC Bioinform..

[18] Sotillo J., Quinzo M., García J.J., Martín-Galiano A.J. (2025). Reverse vaccinology for hookworms: A rational selection of vaccinable antigens against parasitic nematodes. Parasite.

[19] Rathore A.S., Arora A., Choudhury S., Tijare P., Raghava G.P.S. (2023). ToxinPred 3.0: An improved method for predicting the toxicity of peptides. bioRxiv.

[20] Lee S.J., Shin S.J., Lee M.H., Lee M.-G., Kang T.H., Park W.S., Soh B.Y., Park J.H., Shin Y.K., Kim H.W. (2014). A potential protein adjuvant derived from *Mycobacterium tuberculosis* enhances dendritic cell-based tumor immunotherapy. PLoS ONE.

[21] Tarrahimofrad H., Rahimnahal S., Zamani J., Jahangirian E., Aminzadeh S. (2021). Designing a multi-epitope vaccine to provoke robust immune response against influenza A H7N9. Sci. Rep..

[22] Maleki A., Russo G., Palumbo G.A.P., Pappalardo F. (2022). In silico design of recombinant multi-epitope vaccine against influenza A virus. BMC Bioinform..

[23] Khan S., Rizwan M., Zeb A., Eldeen M.A., Hassan S., Rehman A.U., Eid R.A., Zaki M.S.A., Albadrani G.M., Altyar A.E. (2022). Identification of a potential vaccine against *Treponema pallidum* using subtractive proteomics and reverse vaccinology approaches. Vaccines.

[24] Sharma N., Naorem L.D., Jain S., Raghava G.P.S. (2022). ToxinPred2: An improved method for predicting toxicity of proteins. Brief. Bioinform..

[25] Wang Y., Li C., Ban X., Gu Z., Hong Y., Cheng L., Li Z. (2022). Disulfide bond engineering for enhancing thermostability of maltotetraose-forming amylase. Foods.

[26] Kozakov D., Hall D.R., Xia B., A Porter K., Padhorny D., Yueh C., Beglov D., Vajda S. (2017). The ClusPro web server for protein–protein docking. Nat. Protoc..

[27] Comeau S.R., Gatchell D.W., Vajda S., Camacho C.J. (2004). ClusPro: A fully automated algorithm for protein–protein docking. Nucleic Acids Res..

[28] Camacho C.J., Vajda S. (2002). Protein–protein association kinetics and protein docking. Curr. Opin. Struct. Biol..

[29] Case D.A., Aktulga H.M., Belfon K., Cerutti D.S., Cisneros G.A., Cruzeiro V.W.D., Forouzesh N., Giese T.J., Götz A.W., Gohlke H. (2022). AMBER 2022.

[30] Genheden S., Ryde U. (2015). The MM/PBSA and MM/GBSA methods to estimate ligand-binding affinities. Expert Opin. Drug Discov..

[31] Ohara N., Kimura M., Wada N., Yamada T. (1993). Cloning and sequencing of the gene encoding the ribosomal L7/L12-like protein of *Mycobacterium bovis* BCG. Nucleic Acids Res..

[32] Roy K., Kar S., Das R.N. (2015). A Primer on QSAR/QSPR Modeling: Fundamental Concepts.

[33] Badar M.S., Rezaei N. (2022). Molecular Dynamics Simulations: Concept and Applications.

[34] Castiglione F. C-ImmSim: Playing with the immune response. Proceedings of the Sixteenth International Symposium on Mathematical Theory of Networks and Systems (MTNS2004).

[35] Rappuoli R. (2000). Reverse vaccinology. Curr. Opin. Microbiol..

[36] Karagoz A., Tombuloglu H., Alsaeed M., Tombuloglu G., AlRubaish A.A., Mahmoud A., Smajlović S., Ćordić S., Rabaan A.A., Alsuhaimi E. (2023). Monkeypox (mpox) virus: Classification, origin, transmission, genome organization, antiviral drugs, and molecular diagnosis. J. Infect. Public Health.

[37] Hoang Nguyen K.H., Le N.V., Nguyen P.H., Nguyen H.H.T., Hoang D.M., Huynh C.D. (2025). Human immune system: Exploring diversity across individuals and populations. Heliyon.

[38] Cui A., Huang T., Li S., Ma A., Pérez J.L., Sander C., Keskin D.B., Wu C.J., Fraenkel E., Hacohen N. (2024). Dictionary of immune responses to cytokines at single-cell resolution. Nature.

[39] Swetha R.G., Basu S., Ramaiah S., Anbarasu A. (2022). Multi-epitope vaccine for monkeypox using pan-genome and reverse vaccinology approaches. Viruses.

[40] Shantier S.W., Mustafa M.I., Abdelmoneim A.H., Fadl H.A., Elbager S.G., Makhawi A.M. (2022). Novel multi-epitope vaccine against monkeypox virus. Sci. Rep..

