# Supplementary material for: Multiepitope-Based Peptide Vaccine Against A35R Glycoprotein and E8L Membrane Protein of Monkeypox Virus Using an Immunoinformatics Approach
Source: Biology (Basel). 2026 Mar 25;15(7):524. doi: 10.3390/biology15070524 (PMC13072216; doi:10.3390/biology15070524)
Supplement: Supplementary file 1 [file biology-15-00524-s001.zip › biology-4193258-supplementary-revision.pdf]

**Table S1 Physicochemical and Structural properties of target proteins**

| Parameter | A35R Glycoprotein | E8L Membrane Protein |
|---|---|---|
| Alpha helices (%) | 33.70 | 28.62 |
| Extended strands (%) | 18.78 | 20.72 |
| Coils (%) | 47.51 | 50.66 |
| Disulfide bonds | 6 | Not present |
| Disulfide bond positions | Cys36–Cys62, Cys100–Cys109, Cys126–Cys180 | Not applicable |
| Structural prediction tool | trRosetta | trRosetta |
| Model selected | Model 1 | Model 1 |
| Accuracy (%) | 99.7 | — |
| Coverage (%) | — | 76.3 |
| Identity (%) | 86.2 | 34.7 |
| E-value | $1.2 \times 10^{-19}$ | $2 \times 10^{-55}$ |
| Z-score | 25.319 | 20.463 |

**Table S2. Selected MHC Class-I T-cell epitopes for A35R protein with IC-50 value, rank, antigenicity, allergenicity, toxicity, hydrophobicity, GRAVY, PI and solubility**

| A35R MHC Class-1 T-cell epitopes | | | | | | | | | | |
|---|---|---|---|---|---|---|---|---|---|---|
| **Allele** | **Epitope** | **IC-50** | **Rank** | **Allergenicity** | **Antigenicity** | **Toxicity** | **Hydrophobicity** | **GRAVY** | **PI** | **Solubility** |
| HLA-A*03:01 | AIFLQVSDHK | 50 | 0.21 | Non-Allergen | 0.4184 | non-toxic | 25.64 | 0.22 | 7.85 | good water solubility |
| HLA-B*44:03<br>HLA-B*44:02 | KESCNGLYY | 27.98 | 0.02 | | 0.5017 | non-toxic | 15.21 | -0.933 | 6.12 | good water solubility |
| HLA-A*68:01 | VAAASSTHR | 47.14 | 0.41 | | 0.6752 | non-toxic | 3.54 | -0.044 | 10.81 | good water solubility |
| HLA-A*02:01<br>HLA-A*02:06 | FILTAILFL | 8.31 | 0.06 | | 0.5392 | non-toxic | 51.09 | 3.01 | 3.38 | good water solubility |
| HLA-A*02:01<br>HLA-A*02:06 | FILTAILFLM | 37.33 | 0.33 | | 0.4774 | non-toxic | 54.4 | 2.9 | 3.46 | good water solubility |
| HLA-A*32:01<br>HLA-B*15:01<br>HLA-B*58:01 | RSANMSAPF | 5.13 | 0.04 | | 0.9595 | non-toxic | 19.75 | -0.32 | 10.55 | good water solubility |
| HLA-B*35:01<br>HLA-A*30:02 | YVLSTIHIY | 14.21 | 0.07 | | 0.5976 | non-toxic | 32.01 | 1.08 | 7.5 | good water solubility |

**Table S3. Selected MHC Class-I epitopes for E8L protein with IC-50 value, rank, antigenicity, allergenicity, toxicity, hydrophobicity, GRAVY, PI and solubility**

| E8L MHC-I T cell epitopes | | | | | | | | | | |
|---|---|---|---|---|---|---|---|---|---|---|
| **Allele** | **Epitope** | **IC-50** | **Rank** | **Antigenicity** | **Allergenicity** | **Toxicity** | **Hydrophobicity** | **GRAVY** | **PI** | **Solubility** |
| HLA-A*31:01<br>HLA-A*33:01<br>HLA-A*68:01 | FLMSQRYSR | 16.17 | 0.15 | 0.9843 | Non-Allergen | non-toxic | 22.26 | -0.77 | pH 10.96 | Good water solubility |
| HLA-A*33:01<br>HLA-A*68:01<br>HLA-A*31:01 | FMKWLSDLR | 18.89 | 0.05 | 0.6057 | | non-toxic | 37.37 | -0.14 | pH 9.87 | Good water solubility |
| HLA-A*01:01 | ITENYRNPY | 12.32 | 0.03 | 0.8011 | | non-toxic | 15.35 | -1.71 | pH 6.77 | Good water solubility |
| HLA-B*15:01 | RLKTLDIHY | 34.19 | 0.17 | 1.9035 | | non-toxic | 25.92 | -0.56 | pH 9.47 | Good water solubility |
| HLA-B*15:01 | SDLREACFSY | 48.1 | 0.26 | 1.7231 | | non-toxic | 23.26 | -0.35 | pH 3.93 | Good water solubility |

**Figure S1 Multiple sequence alignment of A35R and E8L proteins generated using Clustal Omega**

**Due to the large file size, the figure is provided as a separate PDF.**
