# Supplementary material for: Multiepitope-Based Peptide Vaccine Against A35R Glycoprotein and E8L Membrane Protein of Monkeypox Virus Using an Immunoinformatics Approach
Source: Biology (Basel). 2026 Mar 25;15(7):524. doi: 10.3390/biology15070524 (PMC13072216; doi:10.3390/biology15070524)
Supplement: Supplementary file 1 [file biology-15-00524-s001.zip › Figure-S1.pdf]

Figure S1. Multiple sequence alignment of MPXV A35R protein

n = 20 sequences | length = 181 aa | variable positions = 67, 88

```
Positions 1-60
QJQ40286.1   MMTPENDEEQTSVFSATVYGDKIQGKNKRKRVIGLCIRISMVISLLSMITMSAFLIVRLN
AGR38704.1   ............................................................
AGR38513.1   ............................................................
AGR38322.1   ............................................................
AGR38131.1   ............................................................
AGR37941.1   ............................................................
AGR37751.1   ............................................................
AGR37561.1   ............................................................
AGR37370.1   ............................................................
AGR37179.1   ............................................................
AGR36988.1   ............................................................
AGR36797.1   ............................................................
AGR36606.1   ............................................................
AGR36415.1   ............................................................
AGR36224.1   ............................................................
AGR36033.1   ............................................................
AGR35842.1   ............................................................
AGR35651.1   ............................................................
AGR35460.1   ............................................................
AGR35269.1   ............................................................
Consensus    ************************************************************

Positions 61-120
QJQ40286.1   QCMSANKAAITDSAVAVAAASSTHRKVVSSTTQYDHKESCNGLYYQGSCYILHSDYKSFE
AGR38704.1   ......E....................A................................
AGR38513.1   ......E....................A................................
AGR38322.1   ......E....................A................................
AGR38131.1   ......E....................A................................
AGR37941.1   ......E....................A................................
AGR37751.1   ......E....................A................................
AGR37561.1   ......E....................A................................
AGR37370.1   ......E....................A................................
AGR37179.1   ......E....................A................................
AGR36988.1   ......E....................A................................
AGR36797.1   ......E....................A................................
AGR36606.1   ......E....................A................................
AGR36415.1   ......E....................A................................
AGR36224.1   ......E....................A................................
AGR36033.1   ......E....................A................................
AGR35842.1   ......E....................A................................
AGR35651.1   ......E....................A................................
AGR35460.1   ......E....................A................................
AGR35269.1   ......E....................A................................
Consensus    ****** ******************** ********************************

Positions 121-180
QJQ40286.1   DAKANCAAESSTLPNKSDVLTTWLIDYVEDTWGSDGNPITKTTSDYQDSDVSQEVRKYFC
AGR38704.1   ............................................................
AGR38513.1   ............................................................
AGR38322.1   ............................................................
AGR38131.1   ............................................................
AGR37941.1   ............................................................
AGR37751.1   ............................................................
AGR37561.1   ............................................................
AGR37370.1   ............................................................
AGR37179.1   ............................................................
AGR36988.1   ............................................................
AGR36797.1   ............................................................
AGR36606.1   ............................................................
AGR36415.1   ............................................................
AGR36224.1   ............................................................
AGR36033.1   ............................................................
AGR35842.1   ............................................................
AGR35651.1   ............................................................
AGR35460.1   ............................................................
AGR35269.1   ............................................................
Consensus    ************************************************************

Positions 181-181
QJQ40286.1   T
AGR38704.1   .
AGR38513.1   .
AGR38322.1   .
AGR38131.1   .
AGR37941.1   .
AGR37751.1   .
AGR37561.1   .
AGR37370.1   .
AGR37179.1   .
AGR36988.1   .
AGR36797.1   .
AGR36606.1   .
AGR36415.1   .
AGR36224.1   .
AGR36033.1   .
AGR35842.1   .
AGR35651.1   .
AGR35460.1   .
AGR35269.1   .
Consensus    *
```

Dots indicate identity to the first sequence in each alignment block; letters mark substitutions; asterisks in the consensus row indicate fully conserved positions.

Figure S1. Multiple sequence alignment of MPXV E8L protein

n = 20 sequences | length = 304 aa | variable positions = 19, 213

```
Positions 1-60
QJQ40248.1   MPQQLSPINIETKKAISDARLKTLDIHYNESKPTTIQNTGKLVRINFKGGYISGGFLPNE
AGR38664.1   ..................T.........................................
AGR38473.1   ..................T.........................................
AGR38282.1   ..................T.........................................
AGR38091.1   ..................T.........................................
AGR37901.1   ..................T.........................................
AGR37711.1   ..................T.........................................
AGR37521.1   ..................T.........................................
AGR37330.1   ..................T.........................................
AGR37139.1   ..................T.........................................
AGR36948.1   ..................T.........................................
AGR36757.1   ..................T.........................................
AGR36566.1   ..................T.........................................
AGR36375.1   ..................T.........................................
AGR36184.1   ..................T.........................................
AGR35993.1   ..................T.........................................
AGR35802.1   ..................T.........................................
AGR35611.1   ..................T.........................................
AGR35420.1   ..................T.........................................
AGR35229.1   ..................T.........................................
Consensus    ****************** *****************************************

Positions 61-120
QJQ40248.1   YVLSTIHIYWGKEDDYGSNHLIDVYKYSGEINLVHWNKKKYSSYEEAKKHDDGIIIIAIF
AGR38664.1   ............................................................
AGR38473.1   ............................................................
AGR38282.1   ............................................................
AGR38091.1   ............................................................
AGR37901.1   ............................................................
AGR37711.1   ............................................................
AGR37521.1   ............................................................
AGR37330.1   ............................................................
AGR37139.1   ............................................................
AGR36948.1   ............................................................
AGR36757.1   ............................................................
AGR36566.1   ............................................................
AGR36375.1   ............................................................
AGR36184.1   ............................................................
AGR35993.1   ............................................................
AGR35802.1   ............................................................
AGR35611.1   ............................................................
AGR35420.1   ............................................................
AGR35229.1   ............................................................
Consensus    ************************************************************

Positions 121-180
QJQ40248.1   LQVSDHKNVYFQKIVNQLDSIRSANMSAPFDSVFYLDNLLPSTLDYFTYLGTTINHSADA
AGR38664.1   ............................................................
AGR38473.1   ............................................................
AGR38282.1   ............................................................
AGR38091.1   ............................................................
AGR37901.1   ............................................................
AGR37711.1   ............................................................
AGR37521.1   ............................................................
AGR37330.1   ............................................................
AGR37139.1   ............................................................
AGR36948.1   ............................................................
AGR36757.1   ............................................................
AGR36566.1   ............................................................
AGR36375.1   ............................................................
AGR36184.1   ............................................................
AGR35993.1   ............................................................
AGR35802.1   ............................................................
AGR35611.1   ............................................................
AGR35420.1   ............................................................
AGR35229.1   ............................................................
Consensus    ************************************************************

Positions 181-240
QJQ40248.1   AWIIFPTPINIHSDQLSKFRTLLSSSNHEGKPHYITENYRNPYKLNDDTQVYYSGEIIRA
AGR38664.1   ................................R...........................
AGR38473.1   ............................................................
AGR38282.1   ............................................................
AGR38091.1   ............................................................
AGR37901.1   ................................R...........................
AGR37711.1   ................................R...........................
AGR37521.1   ................................R...........................
AGR37330.1   ............................................................
AGR37139.1   ............................................................
AGR36948.1   ............................................................
AGR36757.1   ............................................................
AGR36566.1   ............................................................
AGR36375.1   ............................................................
AGR36184.1   ............................................................
AGR35993.1   ............................................................
AGR35802.1   ............................................................
AGR35611.1   ............................................................
AGR35420.1   ............................................................
AGR35229.1   ............................................................
Consensus    ******************************** ***************************

Positions 241-300
QJQ40248.1   ATTSPVRENYFMKWLSDLREACFSYYQKYIEGNKTFAIIAIVFVFILTAILFLMSQRYSR
AGR38664.1   ............................................................
AGR38473.1   ............................................................
AGR38282.1   ............................................................
AGR38091.1   ............................................................
AGR37901.1   ............................................................
AGR37711.1   ............................................................
AGR37521.1   ............................................................
AGR37330.1   ............................................................
AGR37139.1   ............................................................
AGR36948.1   ............................................................
AGR36757.1   ............................................................
AGR36566.1   ............................................................
AGR36375.1   ............................................................
AGR36184.1   ............................................................
AGR35993.1   ............................................................
AGR35802.1   ............................................................
AGR35611.1   ............................................................
AGR35420.1   ............................................................
AGR35229.1   ............................................................
Consensus    ************************************************************

Positions 301-304
QJQ40248.1   EKQN
AGR38664.1   ....
AGR38473.1   ....
AGR38282.1   ....
AGR38091.1   ....
AGR37901.1   ....
AGR37711.1   ....
AGR37521.1   ....
AGR37330.1   ....
AGR37139.1   ....
AGR36948.1   ....
AGR36757.1   ....
AGR36566.1   ....
AGR36375.1   ....
AGR36184.1   ....
AGR35993.1   ....
AGR35802.1   ....
AGR35611.1   ....
AGR35420.1   ....
AGR35229.1   ....
Consensus    ****
```

Dots indicate identity to the first sequence in each alignment block; letters mark substitutions; asterisks in the consensus row indicate fully conserved positions.
